# Molecular interactions between parasite and mosquito during midgut invasion as targets to block malaria transmission

**DOI:** 10.1038/s41541-021-00401-9

**Published:** 2021-11-29

**Authors:** Yacob Keleta, Julian Ramelow, Liwang Cui, Jun Li

**Affiliations:** 1 Department of Biological Sciences, Florida International University, Miami, FL 33199 USA; 2 Herbert Wertheim College of Medicine, Florida International University, Miami, FL 33199 USA; 3 College of Public Health, University of South Florida, Tampa, FL 33612 USA; 4 Biomolecular Science Institute, Florida International University, Miami, FL 33199 USA

**Keywords:** Protein vaccines, Parasitic infection

## Abstract

Despite considerable effort, malaria remains a major public health burden. Malaria is caused by five *Plasmodium* species and is transmitted to humans via the female *Anopheles* mosquito. The development of malaria vaccines against the liver and blood stages has been challenging. Therefore, malaria elimination strategies advocate integrated measures, including transmission-blocking approaches. Designing an effective transmission-blocking strategy relies on a sophisticated understanding of the molecular mechanisms governing the interactions between the mosquito midgut molecules and the malaria parasite. Here we review recent advances in the biology of malaria transmission, focusing on molecular interactions between *Plasmodium* and *Anopheles* mosquito midgut proteins. We provide an overview of parasite and mosquito proteins that are either targets for drugs currently in clinical trials or candidates of promising transmission-blocking vaccines.

## Introduction

According to the World Health Organization (WHO), there were approximately 229 million malaria cases worldwide in 2019, resulting in 409,000 deaths. Africa remains the heartland of malaria transmission, accounting for 94% of cases and deaths globally. Five *Plasmodium* species, *P. falciparum, P. vivax, P. malaria, P. ovale*, and *P. knowlesi*, cause malaria in humans, of which *P. falciparum* accounts for approximately 99.7% of malaria cases in Africa^1–3^. *P. falciparum* is also responsible for most malaria-related deaths, especially in children under five years old and in pregnant women^2,4^. Malaria is transmitted to humans by the bite of a female *Anopheles* mosquito. More than 30 species of *Anopheles* mosquitoes in various regions of the globe can transmit malaria. *Anopheles gambiae* is the major vector for the deadliest parasite, *P. falciparum*, in Africa^5,6^. Current malaria control practices rely heavily on clinical case management through a timely diagnosis and effective drug treatment in addition to vector-based measures such as insecticide-treated bed nets and indoor residual sprays of insecticides. However, the emergence of drug-resistant parasites and insecticide-resistant mosquitoes pose increasing challenges to malaria control and elimination^7,8^, demanding the development of innovative technologies.

Vaccine development against malaria has been on the research agenda for malaria control and elimination for decades. Malaria vaccines are generally divided into three categories according to the target stage of the parasite’s life cycle—pre-erythrocytic, blood-stage, and transmission-blocking vaccines (TBV). However, vaccine development against such a complicated eukaryotic parasite is challenging. Currently, the most advanced vaccine, RTS,S, which targets the *P. falciparum* circumsporozoite surface protein (PfCSP), showed <37% protection against *P. falciparum* malaria in phase 3 clinical trials^9–11^. Similarly, the first field trial of a Pfs25-based TBV in Malian adults revealed significant limitations^12^. Given such partial effectiveness of vaccines against single targets at individual stages, it is anticipated that a more efficacious approach would involve the combination of subunit vaccines targeting multiple antigens and multiple stages^13^. This principle is illustrated in a study showing that combining a partially effective pre-erythrocytic vaccine and TBV could synergistically reduce the prevalence of mosquito infections in laboratory mice^14^. With the growing recognition of effective transmission interruption as one key measure for malaria elimination^3,15^, TBVs have received increasing attention. Currently, there are only a few parasite sexual-stage antigens as the “priority” TBV candidates (Table 1), which emphasizes the need for strenuous efforts in antigen discovery to broaden the antigen repertoire^16,17^. These efforts will benefit from a better understanding of the malaria parasite–vector interactions during the midgut invasion process. Here we review recent advances in the identification of key parasite and mosquito molecules that play roles in midgut invasion, and we evaluate their potential for TBV development.

**Table 1:** Malaria TBV candidates for sexual parasite proteins either under preclinical development or in clinical trials.

Protein name	Expression location	Function	Current status	References
Pfs25 (PF3D7_1031000)	Extracellular gametocyte through ookinete	Ookinete survival in the mosquito midgut, penetration of the midgut epithelium, and transformation of ookinetes into oocysts	Phase I	^12,78,79,83,84,86–89,137^
Pfs28 (PF3D7_1014200)	Zygote and ookinete surface	Ookinete invasion in the mosquito midgut and protects ookinetes from midgut enzymes	Preclinical development	^90–92^
Pfs230 (PF3D7_0209000)	Gametocyte and gamete	Gamete–gamete interaction and male gamete fertility	Phase I, about to start phase II	^65,82,93,94,98^
Pfs48/45 (PF3D7_1346700)	Gametocyte and gamete	Essential for parasite fertilization	Preclinical development	^64,103,104,108^
Pfs47 (PF3D7_1346800)	Female gametocyte through ookinetes	Parasitic immune evasion	Preclinical development	^68,69,109–111,130,138^
HAP2 (PF3D7_1014200)	Male gametocytes and microgametes	Fusion and fertilization of gametes	Preclinical development	^71,82,113,115^

**Key**: *PF3D7* *Plasmodium falciparum* gene identifier, *HAP2* HAPLESS2.

## The malaria parasite development in the human host

The life cycle of *Plasmodium* parasites alternates between a female *Anopheles* mosquito and a vertebrate host, involving multiple unique developmental stages that target diverse host cell types^18,19^. In humans, the cycle begins after the injection of sporozoites into the bloodstream by an infected female *Anopheles* mosquito during its blood-feeding. Within 30 min, the sporozoites migrate from the bloodstream to the liver and invade hepatocytes. Inside the liver cells, the now intracellular parasite divides mitotically over several days and eventually develops into a schizont that contains thousands of merozoites^1,20^. Rupture of a schizont releases merozoites into the bloodstream to invade erythrocytes, which initiate the asexual erythrocytic stage of the replication cycle. Inside the red blood cells (RBCs), the parasites undergo schizogony, lasting over 48 h for *P. falciparum*, progressing through the ring, trophozoite, and schizont stages. The schizont subsequently ruptures, releasing 8–36 merozoites to invade other RBCs. This is the stage when the affected patient typically manifests clinical symptoms such as fever and chill in recurring episodes^20–22^.

During the asexual replication cycle within the RBCs, some parasites undergo sexual development and differentiate into male and female gametocytes, a process termed gametocytogenesis^4,23,24^. For *P. falciparum*, the gametocyte commitment generally starts before schizogony, wherein an individual schizont produces a progeny of merozoites that develops into either all asexual or all sexual forms^25–27^. Gametocytogenesis is influenced by a combination of host and environmental stress factors, including anemia^28–31^, drug treatment^29–32^, host immune response^33,34^, and high parasitemia^26^. In vitro, gametocytogenesis can be stimulated by manipulation of the parasite culture, for example by adding red blood cell lysate and lymphocytes together with serum. It was also shown that the nucleic acid synthesis inhibitor Berenil stimulates gametocytogenesis^35–37^.

Recent studies have provided new insights into the mechanisms of sexual commitment and differentiation. For example, sexual commitment in *P. falciparum* has been shown to be governed by the AP2-domain transcription factor PfAP2-G functioning as a master regulator^38–40^. PfAP2-G also regulates early gametocyte development, as it binds to the promoters of many early gametocyte genes such as *Pfs16*, *etramp10.3*, *gexp05*, *Pfg14.744*, and *Pfg14.748*^38^. The *P. falciparum* gametocyte development 1 (Pfgdv1) is an upstream activator of sexual commitment, antagonizing the heterochromatin protein 1-mediated gene silencing of *Pfap2-g*^41–43^. In addition, targeted gene disruption and complementation showed that *P. falciparum* gene implicated in gametocytogenesis (pfgig) also participates in gametocyte commitment^44^. Pfs16, a gametocyte-specific parasitophorous vacuole membrane (PVM) protein, is required for gametocyte development^45,46^. A comprehensive list of genes that are essential for gametocyte development has been reported^24^.

*P. falciparum* gametocytes take 7–10 days to traverse five morphologically distinct stages to reach maturity. Gametocytes in stages I–IV become sequestered in the extravascular spaces of the hematopoietic system such as bone marrow, spleen, brain, heart, and gut, whereas mature gametocytes are released back into the blood circulation^4,47^ where they can be taken up by an anopheline mosquito.

## The *Anopheles* midgut as a trigger for parasite transformation

When a mosquito bites a human, the ingested blood meal regulates mosquito gene expression^48^. Specifically, when the human blood reaches the mosquito midgut, it stimulates the midgut epithelium to secrete factors including 3–13% chitin microfibrils and a variety of proteins^49,50^, which forms a peritrophic matrix (PM)^51^ that surrounds the ingested blood and separates the blood bolus from midgut epithelial cells. The newly formed PM provides another physical barrier that limits the infection of the mosquito by pathogens in the blood meal^52^.

Inside the mosquito midgut, the co-ingested mature gametocytes undergo gametogenesis, gamete fertilization, and ookinete development before penetrating the midgut epithelium and landing at the destination between the midgut endothelium and basal lamina^53,54^. Several mechanistic details of this sequence of events are now understood. Thus, immediately after ingestion, the gametocytes are activated collectively by environmental signals such as a sharp drop in temperature of approximately 5 °C, a rise in pH from 7.4 to 8.0, and a mosquito-derived molecule xanthurenic acid (XA)^55^. Each female extracellular gametocyte forms a single immotile macrogamete, while a male extracellular gametocyte generates up to eight flagella-like microgametes in a process called “exflagellation”. Gametocyte egress from the RBC occurs via an inside‐out fashion with the rupture of the PVM first and the erythrocyte membrane later^56^. Recent studies revealed some of the molecular details of the gametogenesis process. XA activates membrane guanyl cyclases in gametocytes to synthesize cyclic guanosine monophosphate (cGMP), consecutively stimulating the cGMP-dependent protein kinase G (PKG) pathway and significantly increasing the intracellular calcium^57^. A recent CRISPR/Cas9-based functional study demonstrated that an intracellular membrane protein, the gametogenesis essential protein 1 (GEP1), is essential for XA-stimulated gametogenesis regulation in *P. yoelii*^58^. Other signaling molecules identified to be essential for gametogenesis include the male gametocyte-specific kinase PfMAP-2^59^. Cytosolic Ca^2+^ triggers the release of a perforin-like protein PfPLP2, which in turn permeabilizes the erythrocyte membrane, leading to *P. falciparum* gametocyte egress from erythrocytes^60^. *P. falciparum* patatin-like phospholipase 1 (PfPATPL1) with phospholipase A2 (PLA2) activity was found to play a crucial role in gametogenesis by mediating PfPLP2 secretion, gametocyte rounding up, and male gamete exflagellation^61^. Pfs16 was described as an important gene for in vitro male gametocyte exflagellation^46^. In a more recent study, antibodies to Pfs16 resulted in a significant reduction in the number of oocysts. In the same study, five additional parasite proteins including PF3D7_0303900, PF3D7_1204400 (Pfs37), PF3D7_1214800, PF3D7_1239400, and PF3D7_1472800 were found to interact with the mosquito midgut^62^. It has been shown that knocking out Pbg37 (PBANKA_060330) in *P. berghei*, the ortholog of Pfs37, led to a significant reduction in the formation of oocysts^63^. These proteins, along with the previously discussed *Plasmodium* proteins, may serve as novel targets for blocking malaria transmission.

Several *Plasmodium* proteins involved in fertilization and zygote development have been reported. Pfs48/45 is expressed in both male and female gametes, and a gene disruption study of Pfs48/45 revealed that only the male gamete fertility was altered and failed to fertilize the macrogamete^64^. Pfs230 is another critical protein for male fertility and exflagellation^65,66^. A male-specific protein, a disulfide isomerase (PDI-Trans/PBANKA_0820300), is reportedly crucial for fertilization^67^. The female-specific protein P47 is required for the fertility of the macrogametes in *P. berghei*^66^, but not in *P. falciparum*^68^. However, Pfs47 in *P. falciparum* does play a fundamental role in parasitic survival by mediating the evasion of the immune system of the vector^69^. Similarly, the *P. berghei* P47 is also important during ookinete-to-oocyst transition by protecting ookinetes from the complement-like response of mosquitoes^70^. Fertilization starts when a microgamete attaches to a macrogamete and undergoes membrane fusion. HAP2, a conserved protein of protozoan parasites, plants, and algae, has been reported to be essential for *P. berghei* gamete fusion^71^.

Following fertilization, the zygotes undergo meiosis and transform into motile and invasive ookinetes. Expression of over 500 genes in ookinetes is regulated by the AP2 family *Plasmodium* transcription factor AP2-O^72^, which affects ookinete development, motility, midgut penetration, mosquito immunity evasion, and oocyst development initiation^73^. Further studies demonstrated the function of these genes (Table 2), including ookinete surface‐associated proteins like P25 and P28; secretory proteins like chitinase, perforins, PPLP3‐5, and PSOPs^74^; adhesive proteins like the secreted ookinete adhesive protein (SOAP)^75^, the von Willebrand factor A domain‐related protein (WARP)^76^, circumsporozoite- and TRAP-related protein (CTRP)^77^; mobile proteins like glideosome-associated proteins (GAP) and cell traversal protein for ookinetes and sporozoites (CelTOS); and heat shock protein (HSP) 20, 40, 70, and 90^73^. P25 and P28 share multiple functions and are crucial for ookinete development, midgut traversal, and ookinete to oocyst transformation. P25 and P28 are among the top candidates for TBV development^78,79^, a topic that is discussed in greater detail later in this review. The secreted enzymes like chitinase digest the physical barrier of the PM, assisting the ookinete to invade the mosquito midgut^80^. In terms of mobility, *P. yoelii* encoded guanylate cyclase β (GC*β*) is expressed on the ookinetes and localized polarly at the ookinete extrados site (OES) and found to be essential for ookinete gliding. GC*β* contains the N-terminal P4-ATPase-like domain (ALD) and the C-terminal guanylate cyclase domain (GCD), which are required for its polymerization and subsequent ookinete gliding. During ookinete development, CDC50A, as a co-factor of P4-ATPase, stabilizes GC*β*. IMC sub-compartment protein 1 (ISP1) was found to be a crucial molecule for anchoring the GC*β*/CDC50A complex at the OES of mature ookinetes^81^.

**Table 2:** Summary of parasite proteins discovered to be involved in *Anopheles* midgut survival and dissemination.

Protein name	Expression stage	Function	References
Pfgdv1 (PF3D7_0935400)	Gametocytes	Plays a role in transcriptional activation of *PfAP2-G*	^41^
PfAP2-G (PF3D7_1222600)	Gametocytes	Regulation of early gametocyte genes and gametocytogenesis	^38,40^
Pfgig (PF3D7_0935600)	Gametocytes	Regulation of gametocyte commitment	^44^
Pfs16 (PF3D7_0406200)	Gametocytes and gametes	Gametocyte development and male gametocyte exflagellation	^46,62^
PfPLP2 (PF3D7_1216700)	Gametocytes	Key to gametocytes egress from erythrocytes	^60^
PfPATPL1 (PF3D7_0209100)	Gametocytes	Function in gametogenesis by mediating PfPLP2 secretion, gametocyte rounding up, and male gamete exflagellation	^61^
PfMAP-2 (PF3D7_1113900)	Male gametocytes	Gametogenesis and exflagellation	^59^
GEP1 (PF3D7_0515500)	Gametocytes	XA-stimulated gametogenesis regulation	^58^
PDI-Trans/PBANKA_0820300	Male gametocytes	Fertilization and malarial transmission to the mosquito	^67^
Pfs37 (PF3D7_1204400)	Sexual stage-specific protein	May play role in gametogenesis and exflagellation	^62,63^
PF3D7_0303900	??	Phosphatidylethanolamine-binding protein	^62^
PF3D7_1214800	??	unknown	^62^
PF3D7_1239400	??	unknown	^62^
PF3D7_1472800	??	HSP20-like chaperone	^62^
PfCHT1 (PF3D7_1252200)	Ookinetes	Midgut PM invasion	^139,140^
PfCelTOS (PF3D7_1216600)	Ookinetes and sporozoites	Plays a crucial role in establishing malaria infections in both mosquito and vertebrate hosts	^141–143^
SOAP (PF3D7_1404300)	Ookinetes and young oocysts	Interacts with midgut basal laminin and plays a role in oocyst development	^75^
WARP (PF3D7_0801300)	Ookinetes	Midgut invasion	^144^

**Key**: *Pfgdv1* *P. falciparum* gametocyte development 1 gene, *PfAP2-G* *P. falciparum* AP2 transcription factor, *PfMAP-2* *P. falciparum* mitogen-activated protein kinase-2, *GEP1* gametogenesis essential protein 1, *Pfgig* *P. falciparum* gene implicated in gametocytogenesis, *PfPLP2* *P. falciparum* perforin-like protein 2, *PfPATPL1* *P. falciparum* patatin-like phospholipase, *CHT1* chitinase, *PfCelTOS* cell traversal protein for ookinetes and sporozoites, *SOAP* secreted ookinete adhesive protein, *WARP* von Willebrand factor A domain-related protein. **Note**: *Italics* format represents gene name.

## *P. falciparum* TBV candidate proteins

### Pfs25 and Pfs28

The expression of Pfs25 and Pfs28 begins in the extracellular gametocytes within the mosquito vector^82^. Pfs25 is a 25 kDa sexual stage protein mostly expressed on the surface of macrogametes, zygotes, and ookinetes of *P. falciparum* inside the mosquito midgut^83^. Pfs25 was the first protein to progress to a clinical trial; however, the phase 1 trial of this protein, formulated in the adjuvant Montanide ISA51, was found to be reactogenic^84^. Ensuing research focused on combining Pfs25 with other proteins. Fusion of Pfs25 antigen to IMX313, a protein multimerization technology, formulated a nanoparticle with enhanced immunogenicity^85^. A similar study demonstrated stimulation of functional antibody response against malaria infection and transmission in mice by combining Pfs25-IMX313 with RTS, S/AS01^86^. Several studies involving other recombinant products have moved into phase 1 clinical trial, including one involving the conjugation product of Pfs25 linked to a detoxified form of *Pseudomonas aeruginosa* exoprotein A (EPA)^87^, Pfs25-EPA conjugates formulated with Alhydrogel^®^ ^88^, and a chimeric virus-like particle (VLP) containing Pfs25 fused to the alfalfa mosaic virus coat protein^89^. However, the antibody titers in these trials and the effectiveness of transmission-blocking (TB) potential were insufficient. Hence there is a need for an alternative adjuvant.

Similarly, Pfs28 is a 28 kDa protein mainly expressed on the surface of zygotes and ookinetes of *P. falciparum*^90^. Chemical conjugation of Pfs28 to a mutant EPA significantly enhanced immunogenicity in mice immunized with conjugated Pfs28^91^. A 39-kDa chimeric recombinant protein produced by the fusion of Pfs25 and Pfs28 proteins secreted by *Saccharomyces cerevisiae* was found to be more potent than the two candidate proteins alone^92^.

### Pfs230 and Pfs48/45

The expression of the gametocyte antigens Pfs230 and Pfs48/45 starts intracellularly within the human host^82^. Pfs230 and Pfs48/45 are 6-cys family proteins expressed on the surface of gametocytes and gametes of *P. falciparum*^93,94^. Pfs48/45 is expressed by both male and female gametes; however, it is required only for male fertility^64^. Despite their important role in malaria transmission, the development of these TBV candidate antigens has been hampered due to a lack of properly folded recombinant proteins^95^. Although the expression of a full-length recombinant Pfs230 has been hindered by its large size and a large number of disulfide bonds^96,97^, different domains at the N-terminal region of Pfs230 showed TB activities^98^. The Pfs230 N-terminal prodomain, Pfs230C (amino acids 443 to 1132), synthesized using a cell-free system, showed a sufficient complement-dependent malaria TB activity^98^. Antibodies generated against Pfs230C1, another region of the Pfs230 N-terminal (amino acids 443 to 731), significantly reduced the number of oocysts^99^. Pfs230D1+ , a modified form of Pfs230C1 constructed by eliminating the glycosylation property, was found to be homogeneous and more stable than Pfs230C1. Pfs230D1+ also demonstrated higher expression yield and transmission-reducing activity similar to Pfs230C1^100^. Another construct of Pfs230 domain C, 230CMB, produced using a plant-based expression system, showed strong TB activity^101^. In another study, conjugation of Pfs230 with an outer membrane protein complex of *Neisseria meningitidis* was found to have enhanced immunogenicity and functional activity of the Pfs230 protein^102^.

Similar to Pfs230, expression of a full-length recombinant protein Pfs48/45 has been difficult due to its size and complexity, which forced researchers to focus on protein domains. A C-terminal Pfs48/45 expressed in baculovirus demonstrated significant transmission-reducing activity and was found to be homogeneous with respect to its size, conformation, glycosylation, and folding^103^. Several studies showed that chimeras of the 6 C subunit of Pfs48/45 produced in *Lactococcus lactis* are a strong candidate for TBV^104–106^. In another study, monoclonal antibody 85RF45.1 against Pfs48/45 demonstrated a strong reduction of parasite transmission^107^. A recent study reported that a chimeric protein construct of Pfs230 and Pfs48/45, formed by the fusion of the Prodomain of Pfs230 and 6 C fragment of Pfs48/45, showed a higher TB potency than the single proteins alone^108^. This finding bolsters the previous findings with the Pfs25 and Pfs28 fusion protein. All together we can assume that protein chimeras of two or more different proteins can be used to generate antibodies with higher TB activity.

### Pfs47 and HAP2

Pfs47 and HAP2 are other recently identified TBV target proteins. Pfs47 is a member of the 6-cys family of proteins expressed only in macrogametocytes, macrogametes, and ookinetes^94^. Although Pfs47 was found to be dispensable for female gamete fertility, it mediates parasite evasion of the mosquito immune system by suppressing the effect of midgut nitration, a crucial reaction to activate the complement-like system^68,69,109^. While monoclonal antibodies against the full-length Pfs47 protein failed to show TB activity^68^, antibodies targeting a specific region of Pfs47 revealed a significant TB activity^110^. More importantly, conjugation of Pfs47 with AP205 VLP enhanced its immunogenicity and TB activity^111^. Likewise, an in vivo study identified a specific region of *P. berghei* P47 (Pbs47) that offers protection against mosquito immunity. This particular domain has similar immunogenicity to that of Pfs47. Conjugation of the protective antigenic region to a bacteriophage AP205-VLP enhanced immunogenicity and TB activity^112^. A conjugated form of different vaccine candidates has shown higher immunogenicity and better TB activity. Therefore, a conjugated vaccine form of candidate antigens may help resolve the issue of poor immunogenicity during the development of an effective malaria TBV.

On the other hand, PfHAP2, a family member of HAP2 family protein, is expressed only in male gametocytes and activated male gametes^113^. The PfHAP2 recombinant protein-induced IgG antibodies have a significant TB activity^114^. In *P. berghei*, PbHAP2 induced antibodies also showed potent in vitro and in vivo TB activity^113^. A recent study also showed that the anti-*Plasmodium vivax* HAP2 (PvHAP2) antisera significantly reduced the oocyst numbers in mosquito feeding assays using clinical *P. vivax* isolates^115^. Collectively, these findings emphasize that HAP2 is a promising TBV candidate that warrants further investigation and trials.

## Midgut protein interactions affecting *Plasmodium* transmission

**Fig. 1:**
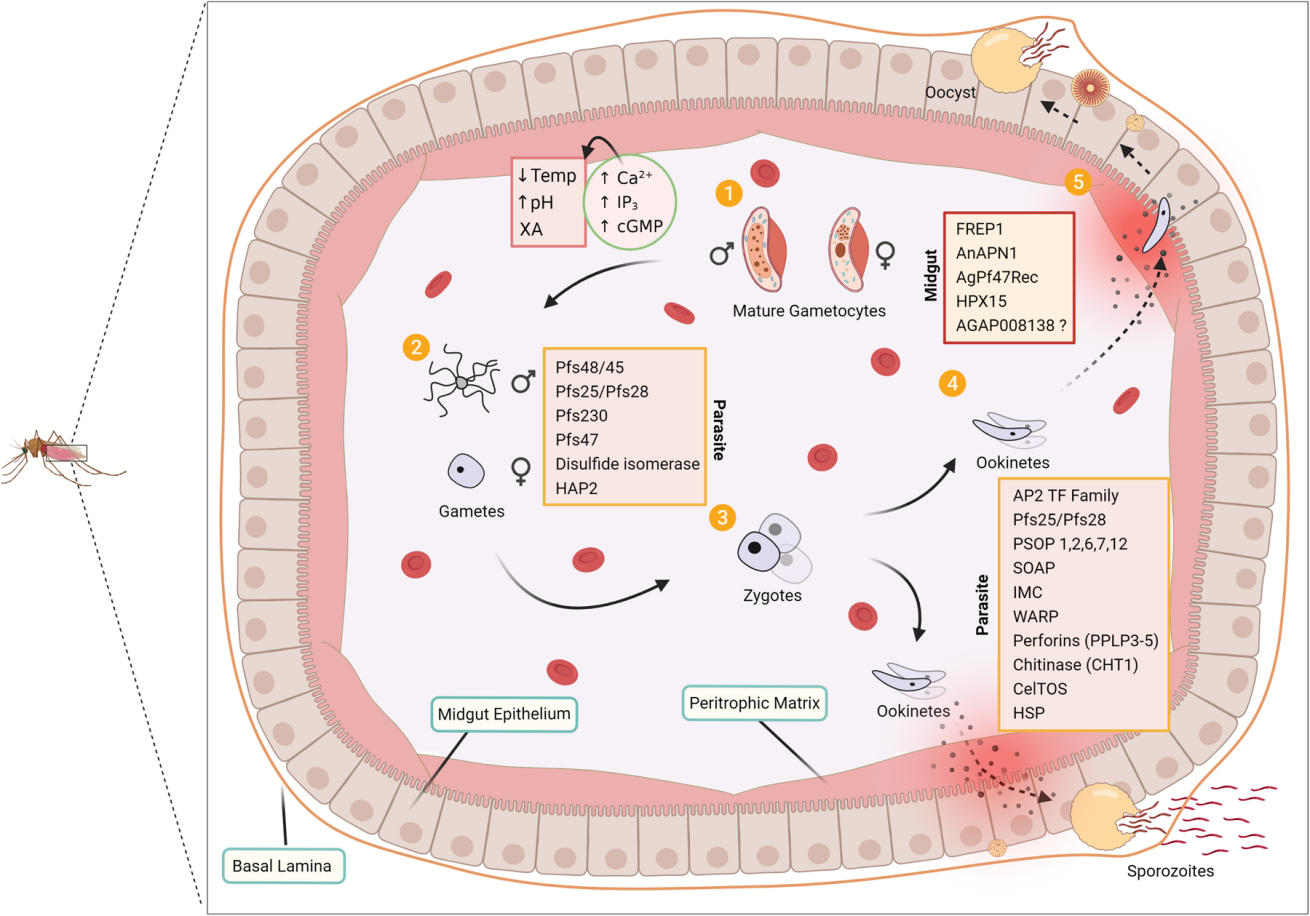
Key proteins responsible for the development of *P. falciparum* parasites inside the *Anopheles* mosquito midgut. **1** The male and female gametocytes ingested during a blood meal egress from the RBCs and become extracellular gametocytes which then develop into male and female gametes via numerous factors such as an increase in pH, a decrease in temperature, and the presence of XA in the midgut environment. This leads to a rise in intracellular Ca^2+^, IP3, and cGMP signaling and to the development of gametes. **2**, **3** The microgametes (male) and the macrogametes (female) increase expression of certain key proteins such as Pfs48/45 and Pfs47 and generate a complete zygote after fertilization. **4** Zygotes develop into motile ookinetes that can penetrate the PM by breaking its chitin structure through chitinases (CHT1), perforins (PPLP3-5), and many other enzymes. **5** Several midgut proteins such as AnAPN1 and FREP1 also help parasites penetrate the peritrophic membrane and midgut epithelium to develop into oocysts at the basal lamina side.

Much of TBV research effort has focused on discovering parasite proteins involved in mosquito invasion. However, recently a concerted effort has been made to elucidate mosquito midgut proteins involved in the parasite invasion process. Essential molecular interactions between the mosquito and the parasite have been discovered, revealing prime TBV candidates such as the AnAPN1, FREP1, and AgPfs47Rec (Fig. 1) as promising TBV targets. Besides these major players, in this section, we will also describe newly discovered mosquito midgut proteins that play a role in the *P. falciparum* transmission process.

### AnAPN1

*Anopheles* alanyl aminopeptidase N (AnAPN1) is considered an important and promising mosquito-based TBV candidate. It is found in the midgut of *A. gambiae* and acts as a ligand for *P. falciparum* and *P. berghei* ookinete invasion^116,117^. The midgut microvilli glycol-conjugates interact with the ookinete and enhances infection of the mosquito^117^. Mosquito midgut glycoproteins are extensively glycosylated with a high proportion of N-linked N-acetylglucosamine (GlcNAc) and N-acetylgalactosamine (GalNAc) terminal oligosaccharides. Glycans, such as GalNAc, and lectins like jacalin, reduce parasite invasion inside the mosquito midgut^118,119^. AnAPN1 at the *A. gambiae* midgut luminal surface is the principal jacalin target and plays an important role in ookinete invasion. The conserved role of AnAPN1 in ookinete invasion of the mosquito midgut explained by the ability of anti-AnAPN1 IgG to strongly inhibit both *P. berghei* and *P. falciparum* development in various mosquito species^117^. Identifying the specific domain of the ligand that interacts with the parasite is important for designing an appropriate TBV.

The AnAPN1 monoclonal antibody, 4H5B7 mAb, showed a potent TB activity at low concentrations^116^. The recombinant AnAPN1 demonstrated acceptable vaccine potency and immunogenicity without immunization-related histopathologies in mice. The AnAPN1 protein has four domains (I to IV), and antibodies against the N-terminal domain-I were found to interfere with the sexual cycle of *P. falciparum* and *P. berghei*^117,120^. Although more research is still required to identify the critical interactions, the resolution of the structure of AnAPN1 is a major advance, which will guide structure-based vaccine design. One such attempt involved using only the relevant domain I of AnAPN1 as the immunogen to reduce unspecific immune stimulation. The domain I construct elicited adequate immune responses in mice, and the IgG antibodies showed potent TB activity^121^.

### FREP1

FREP1 presents another leading candidate for a promising mosquito-based TBV. FREP1 belongs to the fibrinogen-related protein (FREP/FBN) family. In mammals, FREPs play a fundamental role in blood coagulation, while in mosquitoes, they generally act as pattern recognition receptors^122^. Several FREPs activate the mosquito immune system by attaching to external pathogens such as parasites, bacteria, and fungi to activate downstream defense signaling pathways^123,124^. FBN9 and FBN30 were found to inhibit *Plasmodium* infection in midgut epithelial cells. Their functions were illustrated by the increased *Plasmodium* infection of the midgut when FBN9 or FBN30 was knocked down^125,126^. FBN30 is a secreted octameric protein and specifically interacts with the *P. berghei* blood stages and ookinetes, but not sporozoites^127^. Intriguingly, FBN30 was found to interact with the clinically circulating *P. falciparum*, but no interaction occurred with the NF54 *P. falciparum* laboratory strain^127^. Understanding the underlying mechanism for this difference may be helpful for TBV development.

*Anopheles* FREP1 was discovered through an association study using clinically circulating *P. falciparum* and wild-derived *A. gambiae*^126^. Unlike FBN9 and FBN30, which hinder *Plasmodium* infection, FREP1 serves as a molecular anchor in the PM, assisting *P. falciparum* ookinete invasion of the mosquito midgut^128^. The *Plasmodium* α-tubulin-1 protein was found to strongly interact with FREP1 during in vitro studies, and antibodies against α-tubulin-1 blocked malaria transmission inside the mosquito^129^. An increased understanding of the molecular interaction of FREP1 with the α-tubulin-1 may offer another promising TBV candidate. Lastly, it was shown that *FREP1* CRISPR-Cas9 knockout mosquitoes exhibited a profound suppression of parasite infection at both the oocyst and sporozoite stages. Yet, inactivation of the *FREP1* gene inflicted substantial fitness costs on blood-feeding propensity, longevity following a blood meal, fecundity, as well as the rate of egg hatching^53^. A study identified direct interactions of the fibrinogen-like (FBG) domain of FREP1 with *P. falciparum* gametocytes and ookinetes. FBG is highly conserved in different species of *Anopheles* mosquitoes and presents as an effective TBV target against infection from a variety of *Plasmodium* species^124^. The FBG region between amino acids 463 and 677 of *A. gambiae* FREP1 shows more than 90% identity in protein sequence with the FREP1 FBG in 13 anopheline species, suggesting that anti-FBG IgG may potentially block malaria transmission in these 13 mosquito species^5^. Notably, anti-FBG serum showed greater than 81% TB efficiency against *P. falciparum* in *A. gambiae* and ~67% TB efficiency against *P. vivax* in *A. dirus*^23^. This finding further supports that FREP1, specifically the FBG domain, is an ideal TBV target.

### AgPfs47Rec (AGAP006398)

A recent study revealed that Pfs47 mediates immune evasion in mosquitoes through interaction with the *Anopheles* midgut receptor protein AgPf47Rec (AGAP006398)^130^. The interaction allows the parasite to evade the mosquito immune system by disrupting the c-Jun-N-terminal kinase (JNK) signaling pathway and eventually suppressing the effect of midgut nitration, a crucial reaction to activate the mosquito complement-like system^68,69,109,131,132^. Further studies are needed to determine if antibodies against AgPfs47Rec possess TB activity. In addition, the identification of mosquito species-specific AgPfs47Rec and its interaction with various Pfs47 haplotypes provide increasing evidence for a highly specific lock and key model between the ligand and receptor^130^, demonstrating a natural selection of Pfs47 haplotypes in the *P. falciparum* adaptation to different mosquito vectors.

### Other midgut proteins

An increasing number of midgut proteins interacting with the parasite and mediating parasite transmission to mosquitoes have been discovered (Table 3). In a recent study, the *Anopheles* midgut protein AGAP008138 was found to interact with sexual-stage *Plasmodium* parasites and, upon knockdown, increased mosquito susceptibility to *P. falciparum*^133^. Likewise, the evolutionarily conserved heme peroxidase gene HPX15^134^ was found to interact with the *P. falciparum* sexual stages^133^, and knocking down its expression in the midgut after a bloodmeal decreased the oocyst load in *A. gambiae* due to the reduced integrity of the mucin barrier and subsequent activation of midgut immunity pathways^135^. In addition, some immunoglobulin-like secreted proteins such as AGAP002848 and AGAP002851 are pattern-recognition molecules and possibly potent inhibitors of parasite infection^133^. Some structure-related proteins such as AGAP006972 and AGAP006268 are needed to maintain the integrity of the midgut PM and suppress parasitic invasion^133,136^. These newly identified midgut proteins are all worthwhile of further investigation to determine their TB potential and suitability.

**Table 3:** Recognized *Anopheles* midgut proteins involved in malaria transmission.

Protein name	Function	Current status	References
AnAPN1(AGAP004809)	Ligand for ookinete invasion into the midgut	Preclinical development	^116,117^
FREP1(AGAP007031)	Enabling ookinete invasion into the midgut	Preclinical development	^5,128^
HPX15(AGAP013327)	Supports parasite development inside the midgut lumen	Novel candidate	^133–135^
AgP47Rec (AGAP006398)	Plays a critical role in parasite survival by interacting with Pfs47	Novel candidate	^130^
AGAP008138	Solely ookinete invasion facilitating	Novel candidate	^133^
AGAP002848	Protective Immune Niemann Pick Type C2	Novel candidate	^133^
AGAP002851	Protective Immune Niemann Pick Type C2	Novel candidate	^133^
AGAP006972	Unknown	Novel candidate	^133^
AGAP006268	Involved in peritrophic matrix formation	Novel candidate	^133^

**Key:** *AGAP* *Anopheles gambiae* gene identifier, *AgP47Rec* Pfs47 receptor, *AnAPN1* *Anopheles* alanyl aminopeptidase N 1, *FREP1* fibrinogen-related protein 1, *HPX15* Heme peroxidase 15.

## Conclusion

Here we reviewed recent advances in the study of malaria transmission, focusing specifically on molecular mechanisms of interactions between *P. falciparum* and *A. gambiae* mosquito midgut proteins. Overall, TBVs can halt the transmission cycle of the malaria parasite between the mosquito and its vertebrate host. Although TBV development has met many challenges, recent studies have identified novel protein candidates from both the parasite and vector sides. Further investigation of the molecular mechanisms of interaction between the mosquito midgut proteins and the malaria parasite will provide fundamental insights into malaria transmission and accelerate TBV development, ultimately bringing us one step closer to eradicating this devastating disease from this planet.

## Data Availability

All data are provided in the main text, tables and the figure.
